# A Distinctive Human Metabolomics Alteration Associated with Osteopenic and Osteoporotic Patients

**DOI:** 10.3390/metabo11090628

**Published:** 2021-09-16

**Authors:** Shereen M. Aleidi, Eman A. Alnehmi, Mohammed Alshaker, Afshan Masood, Hicham Benabdelkamel, Mysoon M. Al-Ansari, Anas M. Abdel Rahman

**Affiliations:** 1 Department of Biopharmaceutics and Clinical Pharmacy, School of Pharmacy, The University of Jordan, Amman 11942, Jordan; s.aleidi@ju.edu.jo; 2 Department of Botany and Microbiology, College of Science, King Saud University, Riyadh 11451, Saudi Arabia; eman.nahmi@gmail.com (E.A.A.); myalansari@ksu.edu.sa (M.M.A.-A.); 3 Department of Family Medicine and Polyclinic, King Faisal Specialist Hospital & Research Center, Riyadh 11211, Saudi Arabia; shaker@kfshrc.edu.sa; 4 Proteomics Resource Unit, College of Medicine, King Saud University, P.O. Box 2925 (98), Riyadh 11461, Saudi Arabia; afsmasood@ksu.edu.sa (A.M.); hbenabdelkamel@ksu.edu.sa (H.B.); 5 Metabolomics Section, Department of Clinical Genomics, Center for Genomics Medicine, King Faisal Specialist Hospital and Research Centre (KFSHRC), Zahrawi Street, Al Maather, Riyadh 11211, Saudi Arabia; 6 Department of Biochemistry and Molecular Medicine, College of Medicine, Al Faisal University, Riyadh 11533, Saudi Arabia

**Keywords:** metabolomics, bone mineral density (BMD), osteoporosis, osteopenia, mass spectrometry

## Abstract

Osteoporosis is a common progressive metabolic bone disease resulting in decreased bone mineral density (BMD) and a subsequent increase in fracture risk. The known bone markers are not sensitive and specific enough to reflect the balance in the bone metabolism. Finding a metabolomics-based biomarker specific for bone desorption or lack of bone formation is crucial for predicting bone health earlier. This study aimed to investigate patients’ metabolomic profiles with low BMD (LBMD), including those with osteopenia (ON) and osteoporosis (OP), compared to healthy controls. An untargeted mass spectrometry (MS)-based metabolomics approach was used to analyze serum samples. Results showed a clear separation between patients with LBMD and control (*Q*^2^ = 0.986, *R*^2^ = 0.994), reflecting a significant difference in the dynamic of metabolic processes between the study groups. A total of 116 putatively identified metabolites were significantly associated with LBMD. Ninety-four metabolites were dysregulated, with 52 up- and 42 downregulated in patients with LBMD compared to controls. Histidine metabolism, aminoacyl-tRNA biosynthesis, glyoxylate, dicarboxylate metabolism, and biosynthesis of unsaturated fatty acids were the most common metabolic pathways dysregulated in LBMD. Furthermore, 35 metabolites were significantly dysregulated between ON and OP groups, with 11 up- and 24 downregulated in ON compared to OP. Among the upregulated metabolites were 3-carboxy-4-methyl-5-propyl-2-2furanopropionic acid (CMPF) and carnitine derivatives (i.e., 3-hydroxy-11-octadecenoylcarnitine, and l-acetylcarnitine), whereas phosphatidylcholine (PC), sphingomyelin (SM), and palmitic acid (PA) were among the downregulated metabolites in ON compared to OP. This study would add a layer to understanding the possible metabolic alterations associated with ON and OP. Additionally, this identified metabolic panel would help develop a prediction model for bone health and OP progression.

## 1. Introduction

Osteoporosis (OP) is a chronic progressive metabolic bone disease characterized by low bone mineral density (LBMD). The deterioration in microarchitecture and decreased strength of bone increase bone fragility and the risk of fractures [1,2]. OP is an asymptomatic disease, where the patient usually remains undiagnosed until a low-trauma osteoporotic fracture occurs [1]. Mostly, OP manifests later in life, particularly in postmenopausal women [3]. Its prevalence is higher in the aging population of both men and women [4,5,6]. OP has become a serious public health concern worldwide. It negatively impacts patients’ health and quality of life and results in high healthcare expenditures caused by fractures, disability, nursing home placement, and death [6,7].

Several risk factors have been identified as associated with the development of OP. These include age-dependent bone loss, menopause, low body weight, vitamin D and calcium deficiency, use of corticosteroid drugs, and presence of comorbidities such as diabetes mellitus and cardiovascular diseases [1,6,8,9]. Even though OP is commonly known to present itself in aged and postmenopausal women [10], different studies indicate that around one in four men aged 50 and above would develop OP during their lifetime [11].

Central dual-energy X-ray absorptiometry (DXA) scanning is the gold-standard tool for OP diagnosis, and it is based on BMD measurement [10]. DXA results are usually presented as t-scores, calculated in standard deviation (SD), considering the mean BMD of peak bone mass in young adults as the reference [12]. Individuals with a bone mass t-score above −1 are considered normal. In contrast, those with a t-score between −1 and −2.5 are deemed to have low bone mass or osteopenia (ON). In comparison, those with a t-score equal to or less than −2.5 are regarded as having OP [13]. In addition to DXA, quantitative calcaneal ultrasound (QUS) can predict bone fragility fractures depending on measured BMD and bone turnover markers [14]. Despite the wide use of these screening tools for diagnosis, they still lack sensitivity in identifying worsening of disease or patients at risk for progressing to OP [15]. Therefore, accurate, more powerful predictive and prognostic tools for identifying OP are required to prevent bone fragility fractures correlated with LBMD.

Metabolomics is a comprehensive analytical approach that allows qualitative and quantitative analysis of alterations in metabolite levels within biological systems in response to specific stimuli and pathogenesis [16,17]. It provides insight into understanding the mechanisms and progressions underlying various physiological and pathological conditions [18]. Recently, several metabolomics studies have investigated the alterations in the metabolomic profiles associated with BMD. These studies were limited to specific effects in postmenopausal women [19,20,21,22,23,24], animal models [25,26,27,28,29,30], or cultured osteoclastic cells [31]. Although these studies have shown promising results, it is still unclear if they can be generalized to different populations. There is always a need for systemic metabolomics studies in humans that include both genders and aim to identify metabolites associated with LBMD, including both ON and OP conditions while excluding the confounding factors that might affect bone density.

This study aimed to identify the metabolomic profiles associated with LBMD patients compared to healthy controls, considering the effect of several identified confounding factors. Moreover, this study investigates the metabolic alterations associated with specifically bone health in patients with ON and OP. The study findings can add new insight into our understanding of the metabolomic alterations associated with LBMD, which could help identify novel candidate metabolites as possible biomarkers to predict OP progression.

## 2. Results

### 2.1. Clinical Characteristics and Demographics of the Study Population

The clinical characteristics and demographics of the participants are presented in Table 1. According to the DXA data, 31.88% of the participants had normal BMD (control). Similarly, 31.88% were diagnosed with ON, and 36.23% were diagnosed with OP. Most of the participants were females (75.36%), and almost all were in menopause (98%). The prevalence of ON and OP increased with age. No significant difference in body mass index (BMI) existed between the participants. Compared to the control group, ON and OP groups had significantly lower lumbar t-score, femoral t-score, and fasting blood glucose (FBG), triglyceride (TG), and cholesterol levels (Figure S2 and Table 1). Furthermore, the OP group had a significantly lower lumber t-score than the ON group, as shown in Table 1.

### 2.2. The Overall Metabolomic Analysis and Exclusion of Confounder-Associated Metabolites

Initially, 652 metabolites were detected in the patients with LBMD using the LC–MS approach. Metabolomics data were deposited to the EMBL-EBI MetaboLights database with the identifier MTBLS2486. The complete dataset can be accessed at https://www.ebi.ac.uk/metabolights/MTBLS2486 (accessed on 10 September 2021). As shown in Table 1, age was significantly higher in patients with LBMD compared to control participants. In addition, the proportion of females was higher than males in all the study groups.

Furthermore, vitamin D3, calcium, fasting blood glucose, and lipid profiles differed significantly between LBMD patients and control. Therefore, the impact of these confounding factors on the metabolite levels was considered in the ultimate profile. In this study, specific metabolites associated with the different confounding factors, including type 2 diabetes (T2DM), gender, thyroid disease, drugs, BMI, calcium (Ca) levels, vitamin D3 levels, and lipid profiles, were determined and excluded from the overall analysis, as discussed below. The exclusion of the metabolites associated with the confounding factors can enhance the validity of our results in attributing the changes in levels of detected metabolites to the disease-causing process leading to LBMD in the study population.

**Figure 1:**
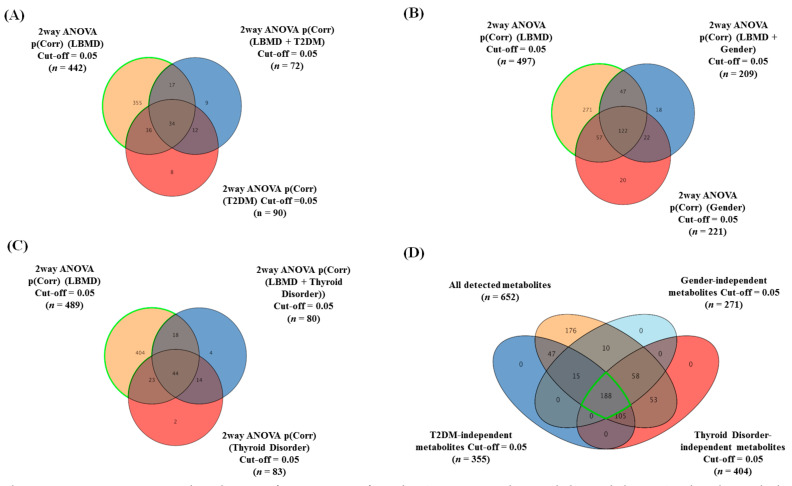
Determination and exclusion of primary confounder (T2DM, gender, and thyroid disease)-related metabolites from the overall detected metabolites. The panels from (**A**–**C**) show Venn diagrams displaying the number of significantly altered metabolites in patients with LBMD, regardless of the effects of primary confounders (T2DM, gender, and thyroid disease), using two-way ANOVA statistical analysis with an FDR-corrected *p*-value cutoff = 0.05. (**A**) The highlighted metabolites depict the number of T2DM-independent metabolites (*n* = 355) from 442 metabolites significantly dysregulated due to the LBMD combined with T2DM. (**B**) The highlighted metabolites depict the number of gender-independent metabolites (*n* = 271) from a group 497 metabolites significantly dysregulated due to LBMD combined with gender. (**C**) The highlighted metabolites depict the number of thyroid disease-independent metabolites (*n* = 404) from a group 489 metabolites significantly dysregulated due to LBMD combined with thyroid disease. (**D**) Venn diagram illustrating overlapping among the confounder (T2DM, gender, and thyroid disease)-independent metabolites with the overall detected metabolites (*n* = 652), considering a corrected *p*-value cutoff = 0.05; 188 metabolites were found to remain after exclusion and were considered as significantly connected to LBMD (primary confounder-independent metabolites).

Two-way ANOVA with FDR-corrected *p*-value (FDRp) cutoff = 0.05 was performed for each group confounder. Groups of 442, 497, and 489 metabolites were significantly dysregulated due to the LBMD combined with the primary confounders (T2DM, gender, and thyroid disorder), respectively (Figure 1). Venn diagram analysis of the two-way ANOVA comparisons (i.e., LBMD, T2DM, LBMD + T2DM) resulted in 355 metabolites associated mainly with LBMD after excluding T2DM and LBMD + T2DM-related metabolites (T2DM-independent, Figure 1A). Similarly, 271 metabolites were associated with LBMD after excluding gender, and LBMD + gender (gender-independent, Figure 1B), and 404 metabolites were associated with LBMD regardless of thyroid disease (thyroid-independent, Figure 1C). Overlapping the T2DM, gender, and thyroid disorder-independent metabolic panels with the overall detected metabolites (*n* = 652) revealed 188 metabolites primarily connected to LBMD (Figure 1D).

**Figure 2:**
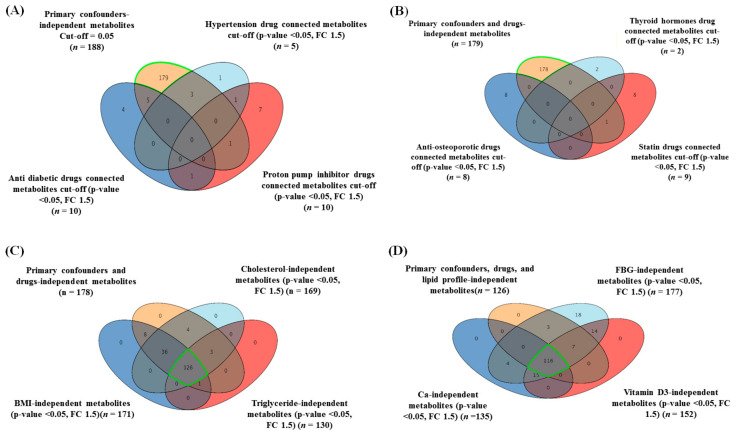
A sequential exclusion of drug- (**A**,**B**) and secondary confounder-related metabolites (**C**,**D**) from the panel of primary confounder-independent metabolites. (**A**,**B**) Venn diagrams illustrating the overlap between the drug-related metabolites and primary confounder-independent metabolites (*n* = 188), using a moderated *t*-test considering fold-change (FC) = 1.5 and cutoff *p*-value < 0.05. A total of 178 metabolites were identified as both drug- and primary confounder-independent metabolites after the exclusion of 10 metabolites. (**C**) Venn diagram illustrating the overlap between metabolites independent of secondary confounders (cholesterol, BMI, and triglycerides (TG)) (*n* = 169, 171, and 130, respectively) and the primary confounder- and drug-independent metabolites identified in (**B**) (*n* = 178), using a moderated *t*-test considering fold-change (FC) = 1.5 and cutoff *p*-value < 0.05. A total of 126 metabolites were identified as metabolites independent from the effects of the primary confounders, drugs, lipid profile, and weight. (**D**) Venn diagram demonstrating further overlap between secondary confounder (FBG, vitamin-D3, and calcium)-independent metabolites (*n* = 177, 152, and 135, respectively) with the primary confounder, drug, lipid profile, and independent weight metabolites identified in (**C**) (*n* = 126), using a moderated *t*-test considering fold-change (FC) = 1.5 and cutoff *p*-value < 0.05. A total of 116 metabolites were identified as significantly associated with LBMD independent of the effect of primary and secondary confounders and drugs.

Furthermore, given that patients’ drugs could influence the metabolic expression, the drug-associated metabolites were determined and excluded from the study’s metabolic profile of primary confounders shown in Figure 1D (*n* = 188 metabolites). Metabolites that were dysregulated by the intake of antidiabetic drugs (*n* = 10), antihypertensive drugs (*n* = 5), proton pump inhibitor (PPI) drugs (*n* = 10), thyroid hormone drugs (*n* = 2), antihyperlipidemic drugs (*n* = 9), and anti-osteoporotic drugs (*n* = 8) were detected using a moderated t-test considering fold-change with cutoffs of 0.05 (*p*-value) and 1.5 (FC), respectively (Supplementary Figure S1). The common drug-related metabolites were excluded from confounder-independent metabolites (*n* = 188) (Figure 1D); therefore, 178 metabolites were identified as confounders and drug-independent panels (Figure 2A,B).

Metabolite levels in human serum are susceptible to specific parameters, including BMI and low-density lipoprotein cholesterol (LDL-C) [32,33]. Additionally, in this study, the triglyceride (TG) levels, fasting blood glucose (FBG), calcium, and vitamin D3 were considered secondary confounders, and their values were integrated into the metabolomics dataset. After excluding these confounder-based metabolites from the identified independent metabolites (*n* = 178), using a moderated t-test (*p*-value < 0.05) and fold-change (FC cutoff 1.5), 116 metabolites were obtained (Figure 2C,D). These putatively identified metabolites (*n* = 116) were significantly associated with LBMD regardless of the primary and secondary confounders and drug-related metabolites.

### 2.3. Metabolomics Profiling of LBMD and Control Groups

**Figure 3:**
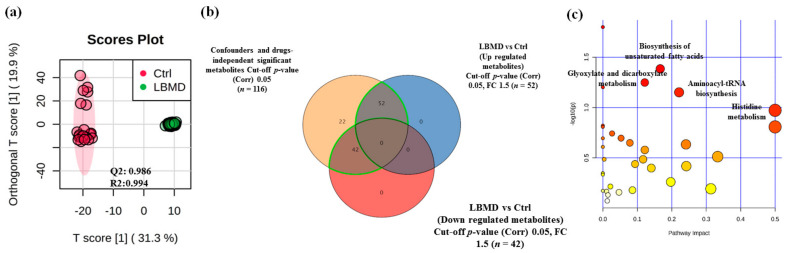
Metabolomics profiling of LBMD and controls. (**a**) An orthogonal partial least squares discriminant (OPLS-DA) analysis of the LBMD versus control metabolomics profile demonstrating clear cluster segregation between both groups (*Q*^2^ = 0.986, *R*^2^ = 0.994). (**b**) Venn diagram showing the dysregulated metabolites detected between LBMD and control groups after applying the fold-change (FC cutoff = 1.5 and *p*-value < 0.05), where 52 and 42 metabolites were up- and downregulated (total 94 metabolites) in LBMD patients compared to control, respectively. (**c**) A pathway analysis plot demonstrating the main pathways involved in the metabolic alterations based on the 94 dysregulated metabolites.

The metabolomics pattern associated with LBMD was examined through an orthogonal partial least squares discriminant analysis (OPLS-DA) score plot. As shown in Figure 3a, a clear separation and grouping between patients with LBMD and control groups was demonstrated (*Q*^2^ = 0.986, *R*^2^ = 0.994), reflecting a significant difference in the metabolic expression study groups.

A binary comparison between metabolite panels of LBMD (independent of confounding metabolites associated with diseases and drugs (*n* = 116) and control groups identified significant dysregulation of 94 metabolites between the two groups (Figure 3b). After applying fold-change analysis (FC = 1.5, and cutoff *p*-value < 0.05) to these 94 metabolites, 52 and 42 metabolites were observed to be up- and downregulated, respectively, in patients with LBMD compared to controls (Figure 3b). Furthermore, metabolic pathway analysis revealed that the most relevant metabolic pathways related to the dysregulation of the identified 94 metabolites included histidine metabolism, aminoacyl-tRNA biosynthesis, glyoxylate and dicarboxylate metabolism, and biosynthesis of unsaturated fatty acids (Figure 3c).

### 2.4. Metabolomics Profiling between ON and OP Groups

**Figure 4:**
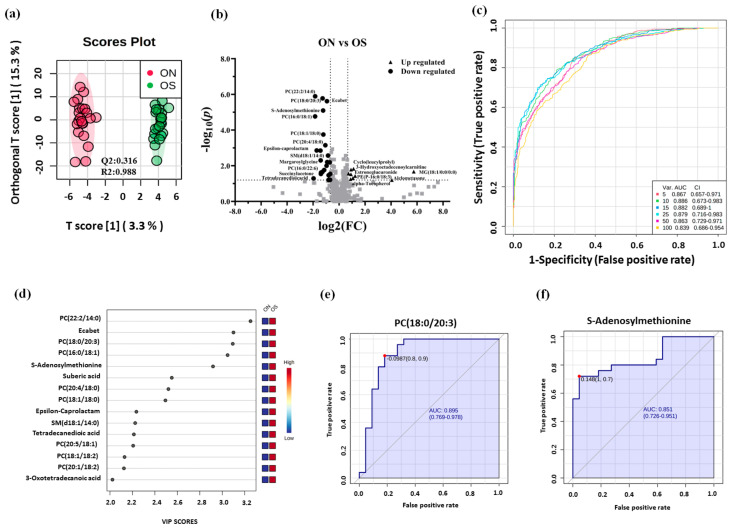
Metabolomics profiling and biomarker evaluation between ON and OP. (**a**) An OPLS-DA analysis of the metabolomics profile of ON versus OP groups showing the separation between these two groups (*Q*^2^ = 0.316, *R*^2^ = 0.988). (**b**) Volcano plot showing the statistically significant dysregulated metabolites (FDR-corrected *p*-value < 0.05, and FC >1.5 or <0.67). The levels of 12 metabolites were upregulated, whereas 24 were downregulated in ON compared to the OP group. (**c**) An exploratory ROC curve was generated by the OPLS-DA model, with AUC values calculated from the combination of five, 10, 15, 25, 50, and 100 proteins. (**d**) Frequency plot showing 15 positively identified metabolites. (**e**,**f**) Representative area under the curve (AUC) for two significantly dysregulated metabolites (*S*-adenosylmethionine, AUC = 0.851, and phosphatidylcholines PC (18:0/20:3), AUC = 0895).

The LBMD patients were further classified into ON and OP groups according to their t-scores. According to the clinical characteristics and demographic data presented in Table 1, there was no significant difference between ON and OP groups in any mentioned parameters, except for lumber and femoral t-scores, along with a slight height difference (Table 1). The metabolomics pattern associated with each group was examined through OPLS-DA (Figure 4a). A relative sample clustering and group separation were noted between ON and OP groups (*Q*^2^ = 0.316, *R*^2^ = 0.988) (Figure 4a), indicating a good metabolic profile and differentially expressed metabolites between these two groups. Volcano plot analysis revealed that 35 metabolites were significantly dysregulated between ON and OP groups considering an FDR-corrected *p*-value < 0.05 and FC > 1.5 or <0.67. Among those dysregulated, 11 were up-and 24 were downregulated in ON compared to the OP group (Figure 4b, Table S1).

Additionally, a multivariate exploratory receiver operating characteristic (ROC) analysis based on the identified significantly dysregulated metabolites between ON and OP (*n* = 35) was generated using OPLS-DA as a classification and feature ranking method. Combining the top 10 metabolites in the exploratory ROC curves indicates the maximum confidence of differentiation and detection of metabolites in the ON versus OP group, with the area under the curve (AUC) = 0.886 (Figure 4c). The significant features of the positively identified metabolites are presented in Figure 4d. Furthermore, representative AUCs for two downregulated metabolites in ON compared to OP (*S*-adenosylmethionine, AUC = 0.851, and phosphatidylcholine PC (18:0/20:3), AUC = 0.895) are shown in Figure 4e,f.

## 3. Discussion

Our study aimed to identify metabolomic profiles associated with LBMD in humans and specifically investigate the metabolic changes associated with ON and OP. Different metabolomics studies of OP in human and animal models were conducted (reviewed in [34]). You et al. were the first research group to investigate the association between the plasma metabolome and BMD in humans using a proton nuclear magnetic resonance spectroscopy (^1^H-NMR) metabolomics approach. They identified significant alterations in four metabolites: lactate, acetone, acetate, and glutamine [19]. Subsequently, several metabolomics studies have highlighted the importance of amino-acid metabolism, lipid metabolism, and bile-acid biosynthesis concerning bone health [23,24,35,36,37,38]. This is the first metabolomics study of LBMD that considers and excludes the effects of several confounding factors that affect bone density to the best of our knowledge. Therefore, our study’s findings can enable a screening of metabolites that would be possible biomarkers related to OP risk prediction or progression. Additionally, it can provide insights into the understanding of metabolomics pathway alterations associated with LBMD.

In this study, patients were classified into normal, ON, and OP groups. An apparent sample clustering and group separation was demonstrated, suggesting the role of LBMD in the revealed distinct serum metabolomics profiles. Several previous studies have enumerated the different factors associated with OP [12,39,40]. It has been shown that T2DM is related to OP and associated with adverse effects on bone formation osteocyte function [39], while antidiabetic drugs might possibly impact OP [40]. Recent studies have shown that thyroid dysfunction has a detrimental effect on bone metabolism, and hyperthyroidism reduces BMD [41].

Furthermore, various in vitro, animal, and clinical studies (reviewed in [42]) investigated the molecular mechanisms of cholesterol-mediated bone deterioration. They demonstrated that hyperlipidemia is negatively correlated with BMD, while treatment with cholesterol-lowering drugs (statin) enhances BMD [42]. Therefore, our study detected and excluded the metabolites related to T2DM, thyroid diseases, hyperlipidemia, gender, drugs, and other confounding factors from the analysis dataset.

The results showed that 116 putatively identified metabolites were independent of the determined confounders and utilized for studying the metabolic expression associated with LBMD. Ninety-four metabolites were dysregulated, with 52 upregulated and 42 downregulated in LBMD compared to controls. Furthermore, investigating the most relevant metabolic pathways associated with dysregulation of these 94 metabolites in LBMD showed histidine metabolism, aminoacyl-tRNA biosynthesis, glyoxylate and dicarboxylate metabolism, and biosynthesis of unsaturated fatty acids (FAs) were the most common with the highest impact value. These findings are consistent with several previous metabolomics studies that emphasized the potential roles of amino-acid, carbohydrate, nucleoside, lipid, and FA metabolism in bone health [23,24,36]. In addition, urinary metabolomics profile analysis of pre-and postmenopausal OP indicated that amino-acid metabolism (such as taurine and β-alanine) and energy metabolism pathways were related to LBMD [35].

Furthermore, the results indicated that 35 metabolites were significantly dysregulated between ON and OP groups, with 11 up- and 24 downregulated in ON compared to OP. Among the upregulated metabolites were 3-carboxy-4-methyl-5-propyl-2-2furanopropionic acid (CMPF) and carnitine derivatives (i.e., 3-hydroxy-11-octadecenoylcarnitine and l-acetylcarnitine), whereas phosphatidylcholine (PC), sphingomyelin (SM), and palmitic acid (PA) were among the downregulated metabolites in ON compared to OP.

CMPF is a metabolite derived from furan fatty acids; it is not de novo synthesized in humans but found in marine animals [43]. It is detected in human urine [44]. In addition, it is known to be accumulated in the serum of patients with chronic kidney disease (CKD) as a uremic toxin [45,46]. Recent metabolomic work has indicated that CMPF levels may be associated with LBMD in postmenopausal women [38]. In line with this, our results showed that levels of CMPF were negatively associated with LBMD. However, further studies are required to prove this hypothesis in both genders; notably, the CMPF serum levels could be altered according to the patient dietary choices [47].

In LBMD, there is a decrease in osteoblast differentiation and mineralization and an increase in osteoclast activity, which results in ON and more severe progressive OP [48]. The decreased osteoblast activity in OP is possibly related to the decrease in energy production [49]. Carnitines are acyl group transporters from the cytoplasm to mitochondria for energy production and FA metabolism [50]. Long-chain acetylcarnitine esters transport fatty acyl moieties across the inner mitochondrial membrane into the mitochondrial matrix for β-oxidation and energy production [50]. Our study results indicated that levels of carnitine derivatives (3-hydroxy-11-octadecenoylcarnitine and l-acetylcarnitine) were significantly downregulated in the OP group compared to ON (upregulated in ON compared to OP). 3-Hydroxy-11-octanecenoylcarnitine is one of the acylcarnitines believed to have about 1000 molecules expressed in the human body [51]. It is considered one of the long-chain acylcarnitines formed through esterification with long-chain fatty acids obtained from the diet [51]. In particular, 3-hydroxy-11*Z*-octadecenoylcarnitine is elevated in the blood or plasma of individuals with chronic fatigue syndrome [51], mitochondrial trifunctional protein deficiency [52], and psoriasis [53]. Previous metabolomic studies showed that l-carnitine [25] and carnitine derivatives such as glutarylcarnitine [37], acetylcarnitine [37], and isovaleryl-carnitine [24] were significantly associated with BMD. Therefore, a change in carnitine levels is a possible indicator of bone health and disease progression.

Interestingly, the biosynthesis of unsaturated FAs was among the significantly altered metabolic pathways according to the dysregulated metabolic panel. In detail, different FAs, whether free or incorporated in PC and SM structures, were dysregulated between ON and OP. Previous evidence indicated that FAs have a vital role in stimulating osteoclastogenesis and osteolysis [28,54]. It has been shown that the saturated FA, palmitic acid (PA, 16:0), promotes receptor activator of NF-κB ligand (RANKL)-stimulated osteoclastogenesis and can also induce osteoclast differentiation even in the absence of RANKL [54]. In addition, high PA levels in an animal model negatively affected osteoblast function and bone health [55].

Moreover, levels of metabolites containing PA, such as phosphatidylcholine (PC (16:0/18:3)), were associated with a higher prevalence of LBMD in postmenopausal Chinese women [38]. Consistent with these previous findings, our metabolomics analysis revealed that PA, PCs, and SMs were significantly changed between ON and OP groups, and their levels were negatively associated with BMD. This suggests that FAs and some lipid classes are closely related to the pathogenesis of the bone disease. Therefore, further lipidomics analysis is required to investigate the changes in lipid classes associated specifically with ON and OP.

In this study, confounder-connected metabolites were excluded from the BMD- related profile, using an analytical approach that would enhance the validity of the results by eliminating the effect of confounders on the metabolomics profile. The number of putative metabolites associated with BMD was identified. However, validation of these putative metabolites is required using an independent cohort from different backgrounds. In addition, the sample size in this study was relatively small. Recruiting control participants was challenging since looking for healthy, medically free, and age-matched participants with a normal DXA scan was difficult.

## 4. Materials and Methods

### 4.1. Patients

This exploratory cohort study involved 69 participants recruited from December 2017–January 2019 from the OP Clinic at King Faisal Specialist Hospital and Research Center (KFSHRC), Riyadh, Saudi Arabia. Lumbar and femoral t-scores were measured using a DXA scan. According to the WHO diagnostic criteria and the BMD t-score, participants were categorized into three groups. Those with a BMD t-score less than −2.5 were considered the osteoporotic group (OP, *n* = 25), those with a t-score between −1 and −2.5 were considered the osteopenic group (ON, *n* = 22), and those with a t-score greater than −1.0 were the healthy control group (Ctrl, *n* = 22). Both ON and OP groups were initially considered together as the LBMD group. Inclusion criteria were males and females ≥50 years old with clinical confirmation of either ON or OP diagnosis. Participants under 50 and those diagnosed with concomitant hyperparathyroidism, chronic infectious arthritis, chronic lung disease, hepatic disease, cardiovascular diseases, and renal failure were excluded from this study. In addition, patients on medications such as glucocorticoids or hormonal replacement (estrogen and androgen therapy) were excluded. Participants’ demographic and clinical data were collected using an approved questionnaire from the primary physician (M.S.).

### 4.2. Metabolomic Analysis

Serum samples were analyzed using label-free untargeted liquid chromatography-mass spectrometry (LC–MS), as described elsewhere [56]. Briefly, 300 μL of cold acetonitrile and 10 μL of 2.8 mg/mL dl-*o*-chlorophenyl alanine internal standard were added to a 100 μL serum sample, followed by vortex mixing for 30 s. The samples were allowed to stand for 1 h at −20 °C to enhance the protein precipitation, centrifuged at 15,000 rpm at 4 °C for 15 min, and dried in a vacuum concentrator. Dry residue was re-dissolved in methanol/water in a ratio of (1:1) before LC–MS analysis.

The separation was performed by an Ultimate 3000LC combined with Q Exactive MS (Thermo Fisher Scientific, Carlsbad, CA, USA) and screened with electrospray ionization (ESI)-MS. The extracted metabolites were chromatographed using an ACQUITY UPLC HSS T3 (100 × 2.1 mm, 1.8 μm) column. The mobile phase was composed of solvent A (0.05% formic acid–water) and solvent B (ACN) with a gradient elution (1–16 min, 95–5% A; 16–18 min, 5% A; 18–19 min, 5–95% A; 19–20 min, 95–95% A). The flow rate of the mobile phase was 300 µL/min. The column temperature was maintained at 40 °C, and the sample manager temperature was set at 4 °C. Mass spectrometry parameters in ESI+ and ESI− modes were kept as follows: heater temperature, 300 °C; sheath gas flow rate, 45 arb; aux gas flow rate, 15 arb; sweep gas flow rate, 1 arb; spray voltage, 3.0 kV; capillary temperature, 350 °C; S-Lens RF level, 30%.

Metabolomics and lipidomics in this manuscript were reported according to the most acceptable guideline for metabolomics identification and annotation [57]. The precursor and product ion spectra were matched to the aligned feature with a precursor tolerance (*m*/*z*) and retention time tolerance of 5 ppm and 15 s, respectively. The identification was performed using several databases such as Human Metabolome Database (www.hmdb.ca (accessed on 19 August 2021)), METLIN (www.metlin.scripps.edu (accessed on 20 March 2020)), and Mass Bank (www.massbank.jp (accessed on 20 March 2020)). The library search was obtained at a precursor *m*/*z* tolerance of 5 ppm combined with a 500 MS/MS score threshold and isotope pattern match of 100 (mSigma).

The unidentified features were putatively identified by mass match with an *m*/*z* tolerance of 5.0 ppm. Some lipid molecules detected in this study had isomeric or isobaric structures. The identification possibilities for each detected feature within the *m*/*z* tolerance of 5.0 mDa were ranked by the filtering and scoring approach described previously [58,59]. Isomeric or isobaric identifications that had elution within the expected retention time range for each lipid class or subclass, the most likely adduct form, the smallest *m*/*z* error, and an even number of carbons in fatty acyl side chains were selected as the most likely identification for the choice of lipid subclass. Other isomeric and isobaric possibilities that passed the retention time and adduct filters were kept but not considered to determine lipid subclass.

The putatively identified lipids (MS/MS or mass match) were divided into subclasses and categories following the classification system proposed by the International Lipid Classification and Nomenclature Committee (ILCNC), the LipidMaps database, and the Lipidomics Standard Initiative (https://lipidomics-standards-initiative.org (accessed on 1 April 2020)) [60]. Abbreviations of lipid classes and subclasses are defined in Table S1. The positions of double bonds and the stereospecific configuration of glycerol derivatives were not determined in this study.

### 4.3. Statistical Analysis

MetaboAnalyst version 5.0 (McGill University of Montreal, Montreal, QC, Canada) was used to process the study MS metabolomics data [61]. The raw data were normalized to the total sample median, log-transformed, and Pareto-scaled to provide all the Gaussian-distributed signals. A univariate analysis using a volcano plot analysis was performed for each binary comparison to identify significantly differentially expressed metabolites based on a fold-change criterion greater than 1.5 or less than 0.67 with a false discovery rate (FDR)-adjusted *p*-value less than 0.05. The *x*-axis on the volcano plot represents the fold-change (FC) between two comparison groups, while the *y*-axis represents the *p*-value. Multivariate analysis (orthogonal partial least squares discriminant analysis (OPLS-DA)) was carried out to identify any clustering or separation between the compared datasets.

For statistical analysis among the groups, analysis of variance (ANOVA) using a post hoc Tukey analysis method, with multiplicity-adjusted *p*-values for each comparison, was used. This analysis seemed best to reduce the probability of making a type 1 error. As seen in our cohorts, it supports the testing of pairwise differences due to the unequal group sizes among the experimental and the control groups. A Pearson similarity test, hierarchical clustering combined with heat maps, and Venn diagram analyses including the two-way ANOVA were performed between the study groups using Multiple Professional Profiler (MPP) software (Agilent In., Santa Clara, CA, USA). According to this study’s metabolic dysregulation, the list of significantly identified metabolites was entered into the pathway analysis module for significant pathway identification. The potential biomarkers were evaluated for their sensitivity and specificity to show bone health using receiver operating characteristic (ROC) curves based on the OPLS-DA method (MetaboAnalyst software version 5.0, Alberta, AB, Canada).

## 5. Conclusions

This study presented a metabolomics pattern associated with LBMD compared to control participants with normal BMD using an analytical approach that excluded the effect of several identified confounding factors. In addition, metabolomic analysis between ON and OP groups identified several dysregulated metabolites (either up- or downregulated) in ON compared to OP, which are sensitive to determining the bone density status in these patients. The findings of this study can add a layer of information to understanding the possible metabolic alterations associated with LBMD.

## Figures and Tables

**Table 1:** Clinical characteristics and demographics of the study population (*n* = 69).

	Ctrl		ON		OP	
Total *n* (%)	22 (31.88)		22 (31.88)		25 (36.23)	
Parameters	Mean	SEM	Mean	SEM	Mean	SEM
Age (years)	54.82	1.03	64.64 ^§^	1.72	66.16 ^§^	1.78
Gender (F/M)	(13/9)	-	(15/7)	-	(24/1)	-
Menopause * (yes/no)	(13/0)	-	(14/1)	-	(24/0)	-
Weight (kg)	85.13	3.63	74.21	3.88	69.23 ^§^	2.86
Height (cm)	162.22	0.02	157.11 ^ǂ^	0.021	150.68 ^§^	0.01
BMI (kg/m^2^)	32.21	1.1	30.38	1.84	30.70	1.4
Lumbar t-Score	0.29	0.24	−1.25 ^§,ǂ^	0.21	−2.62 ^§^	0.12
Femoral t-Score	0.34	0.29	−1.51 ^§,ǂ^	0.14	−1.93 ^§^	0.13
FBG (mmol/L)	10.2	1.16	6.08 ^§^	0.39	5.87 ^§^	0.41
HDL (mmol/L)	1.00	0.80	1.47 ^§^	0.12	1.42 ^§^	0.09
TG (mmol/L)	1.85	0.15	1.23 ^§^	0.11	1.127 ^§^	0.08
Cholesterol (mmol/L)	5.51	0.23	4.47 ^§^	0.19	4.27 ^§^	0.29
Calcium (mmol/L)	2.24	0.026	2.37 ^§^	0.025	2.33 ^§^	0.02
Albumin (g/L)	37.65	1.14	41.98 ^§^	2.0	42.75 ^§^	0.86
Vitamin D 25 hydroxy (nmol/L)	68.32	7.39	77.64	3.3	86.57	6.05

Abbreviations; ON: osteopenic, OP: osteoporotic, BMI: body mass index, FBG; fasting blood glucose, LDL; low-density lipoprotein, HDL; high-density lipoprotein, TG; triglycerides. Data are presented as the mean ± standard error of the mean (SEM); * menopause status in females; ^§^ *p*-value < 0.05 vs. control group; ǂ *p*-value < 0.05 vs. OP group.

## Data Availability

Metabolomics data were deposited to the EMBL-EBI MetaboLights database with the identifier MTBLS2486. The complete dataset can be accessed at https://www.ebi.ac.uk/metabolights/MTBLS2486 (10 September 2021).

## Supplementary Materials

The following are available online at https://www.mdpi.com/article/10.3390/metabo11090628/s1: Figure S1. Determination of drug-related metabolites (drug-dependent) from the overall detected metabolites; Figure S2: Comparisons of clinical characteristics and demographic data between ON and OP groups, Table S1: List of the dysregulated metabolites (11 up- and 24 downregulated in ON compared to OP).
