# Supplementary material for: A Distinctive Human Metabolomics Alteration Associated with Osteopenic and Osteoporotic Patients
Source: Metabolites. 2021 Sep 16;11(9):628. doi: 10.3390/metabo11090628 (PMC8466514; doi:10.3390/metabo11090628)
Supplement: Supplementary file 1 [file metabolites-11-00628-s001.zip › 7 th final version supplemntary figures 16.8.2021.pdf]

Supplementary Figures

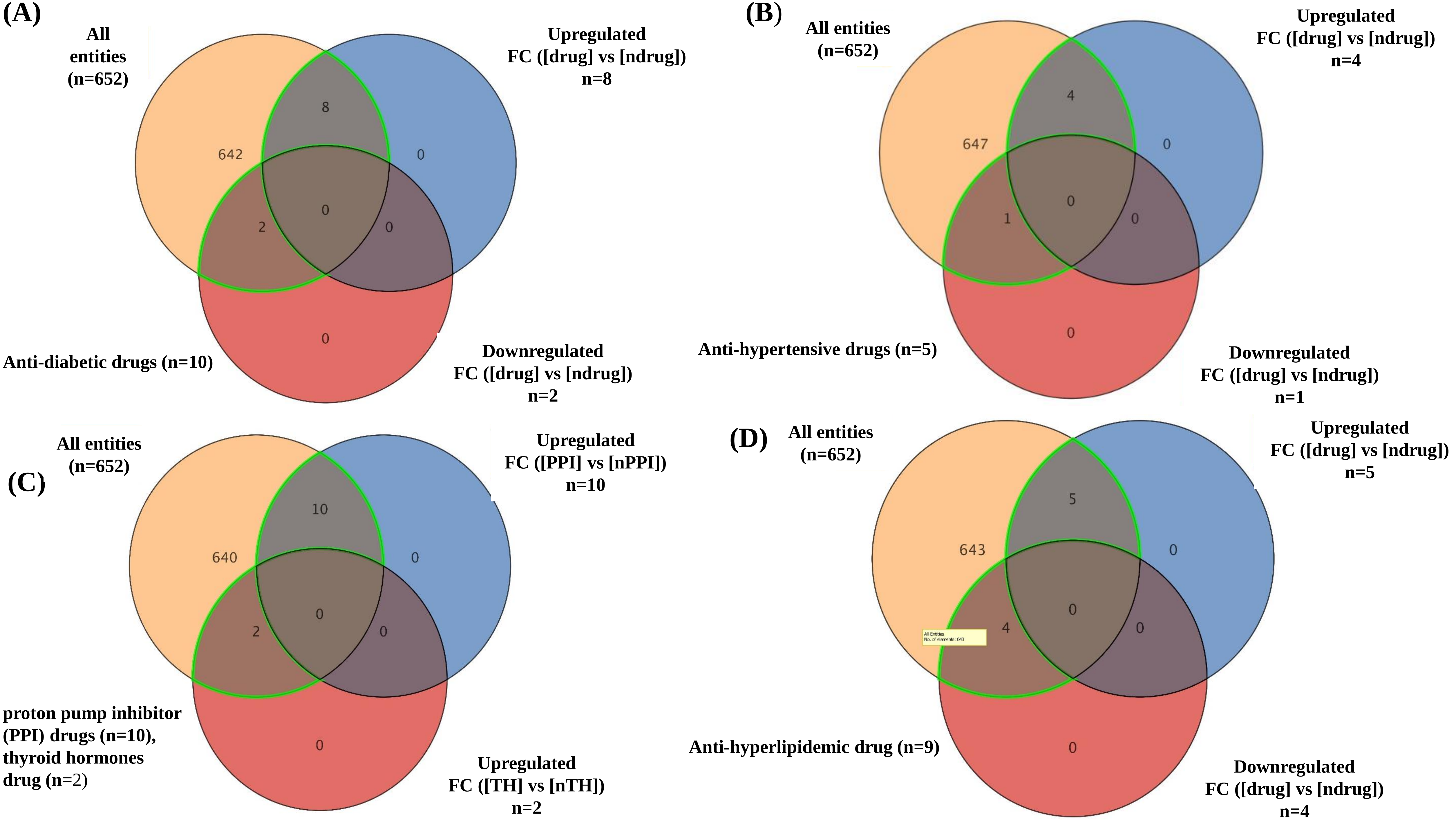

(A)
All entities (n=652)
Upregulated FC ([drug] vs [ndrug]) n=8
642
8
0
2
0
0
0
Anti-diabetic drugs (n=10)
Downregulated FC ([drug] vs [ndrug]) n=2
(B)
All entities (n=652)
Upregulated FC ([drug] vs [ndrug]) n=4
647
4
0
1
0
0
0
Anti-hypertensive drugs (n=5)
Downregulated FC ([drug] vs [ndrug]) n=1
(C)
All entities (n=652)
Upregulated FC ([PPI] vs [nPPI]) n=10
640
10
0
2
0
0
0
proton pump inhibitor (PPI) drugs (n=10), thyroid hormones drug (n=2)
Upregulated FC ([TH] vs [nTH]) n=2
(D)
All entities (n=652)
Upregulated FC ([drug] vs [ndrug]) n=5
643
5
0
4
0
0
0
All Entities
No. of elements: 643
Anti-hyperlipidemic drug (n=9)
Downregulated FC ([drug] vs [ndrug]) n=4

(E)

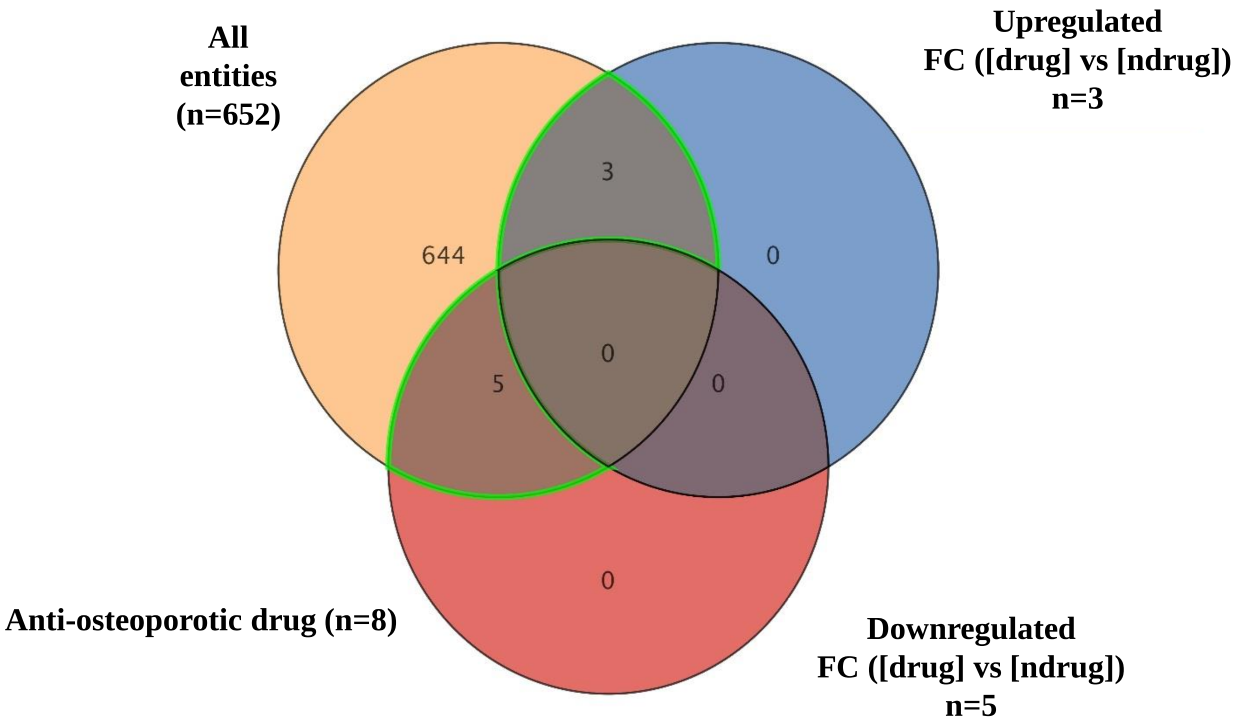

Supplementary Figure S1 : Determination of drug-related metabolites (drug-dependent) from the overall detected metabolites

The panels **(A-E)** show Venn diagrams analysis to detect the dysregulated metabolites as an effect of different drugs intake, using moderate t-test and considering fold change (FC 1.5) and p-value <0.05. **(A)** Metabolites dysregulated (n=10) as an effect of anti-diabetic drugs (8 upregulated and 2 downregulated), **(B)** Metabolites dysregulated (n=5) as an effect of anti-hypertensive drugs (4 upregulated and 1 downregulated), **(C)** Metabolites dysregulated as an effect of proton pump inhibitor (PPI) drugs (n=10) and thyroid hormones drugs (n=2), all were upregulated, **(D)** Metabolites dysregulated (n=9) as an effect of anti-hyperlipidemic (statin) drug (5 upregulated and 4 downregulated) (E) Metabolites dysregulated (n=8) as an effect of anti-osteoporotic drug (3 upregulated and 5 downregulated)

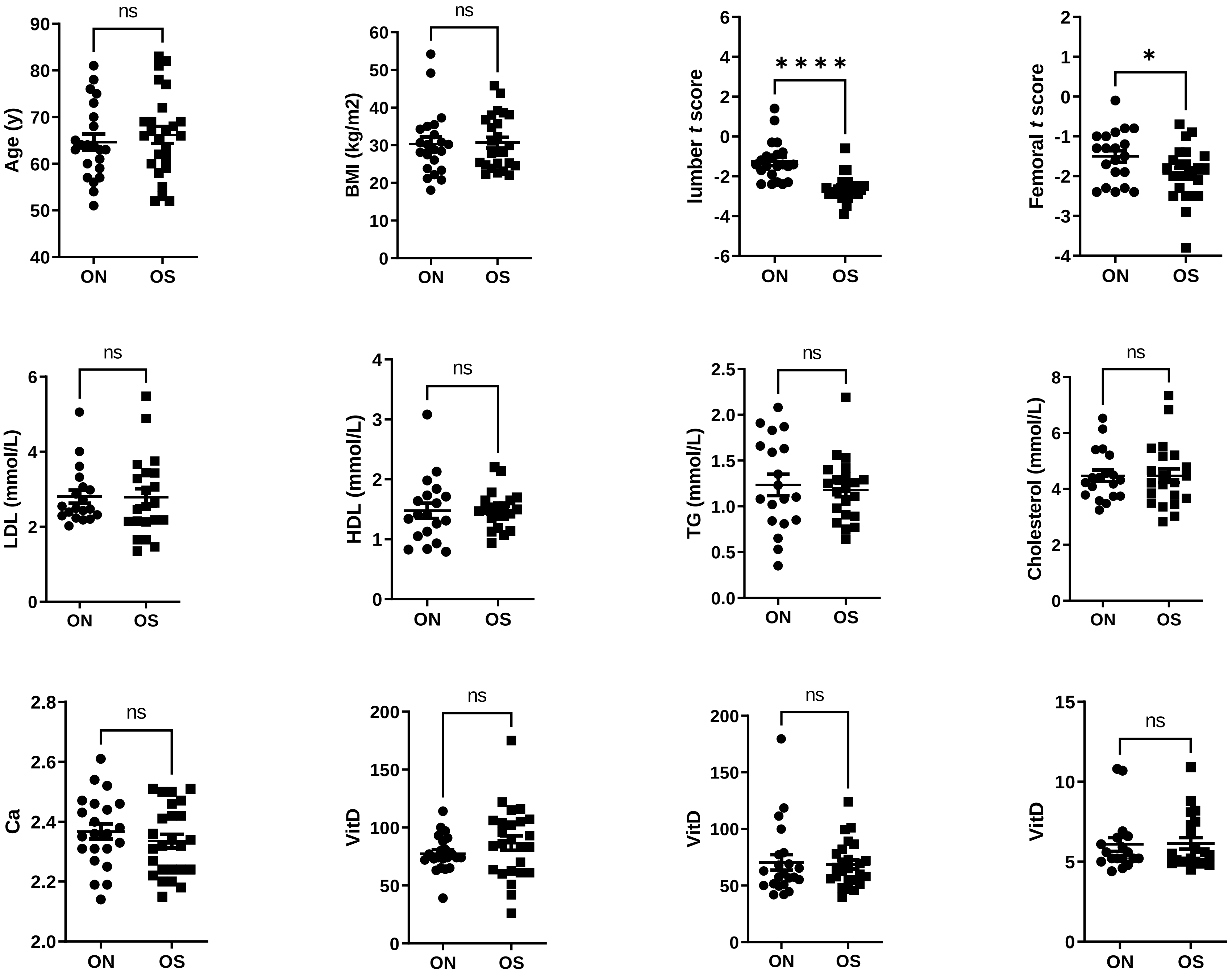

Supplementary Figure S2: Comparisons of clinical characteristics and demographic data between ON and OP groups.
