# Supplementary material for: A Distinctive Human Metabolomics Alteration Associated with Osteopenic and Osteoporotic Patients
Source: Metabolites. 2021 Sep 16;11(9):628. doi: 10.3390/metabo11090628 (PMC8466514; doi:10.3390/metabo11090628)
Supplement: Supplementary file 1 [file metabolites-11-00628-s001.zip › Supplementary Table S1.pdf]

Supplementary Table S1: List of the dysregulated metabolites (11 Up- and 24 down- regulated in ON compared to OP)

ID	RT [min]	Compound name	HMDB ID	Formula	Molecular weight (g/mole)	Monoisotopic Mass	m/z error (ppm)	ESI charge	log2(FC)	-log10(P)
2968	10.127	PC(16:0/18:1)	HMDB0007972	C42H82NO8P	759.5779	759.5778	4	+	-1.851	4.766
3052	10.163	PC(22:2/14:0)	HMDB0008590	C44H84NO8P	785.5953	785.5935	2	+	-1.844	5.8912
2953	10.164	PC(20:1/14:1)	HMDB0008296	C42H80NO8P	757.5645	757.5622	3	+	-1.7254	2.8554
81	2.798	Epsilon-caprolactam	HMDB0062769	C6H11NO	113.0847	113.0841	6	+	-1.4331	2.8508
2914	10.619	SM(d18:0/18:1)	HMDB0012089	C41H83N2O6P	730.6006	730.5989	2	+	-1.4183	2.301
3109	10.762	PC(16:0/22:6)	HMDB0007991	C46H80NO8P	805.5629	805.5622	1	+	-1.399	1.5414
759	6.684	Tetradecanedioic acid	HMDB0000872	C14H26O4	258.1824	258.1831	3	-	-1.3877	1.6204
2857	10.785	SM(d18:1/16:1)	HMDB0240613	C39H77N2O6P	700.5538	700.5519	3	+	-1.2828	1.6563
3148	10.619	PC(18:0/20:3)	HMDB0008046	C46H86NO8P	811.6102	811.6091	1	+	-1.2724	5.7782
1663	10.164	S-Adenosylmethionine	HMDB0001185	C15H23N6O5S	399.1386	399.1451	16	-	-1.2273	5.0956
3063	10.163	PC(18:1/18:0)	HMDB0008102	C44H86NO8P	787.6082	787.6091	1	+	-1.2141	3.749
3040	10.761	PC(18:1/18:2)	HMDB0008105	C44H82NO8P	783.5792	783.5778	2	+	-1.146	1.7579
3133	10.162	PC(20:4/18:0)	HMDB0008464	C46H84NO8P	809.5948	809.5935	2	+	-1.0602	3.147
3175	10.752	PC(18:1/22:6)	HMDB0008123	C48H82NO8P	831.5773	831.5778	1	+	-0.96515	1.9995
1550	10.165	Ecabet	HMDB0015613	C20H28O5S	380.1647	380.1657	3	-	-0.93329	5.6191
925	10.752	Vaccenic acid	HMDB0003231	C18H34O2	282.2552	282.2559	3	-	-0.91683	2.206
2810	10.164	SM(d18:1/14:0)	HMDB0012097	C37H75N2O6P	674.538	674.5363	3	+	-0.85045	2.5723
1453	9.14	N-arachidonoylglycine	HMDB0005096	C22H35NO3	361.2613	361.2617	1	-	-0.80395	1.4671
1236	9.802	Margaroylglycine	HMDB0013246	C19H37NO3	327.2769	327.2773	1	-	-0.71817	2.1874
173	3.269	Succinylacetone	HMDB0000635	C7H10O4	158.0564	158.0579	10	-	-0.6816	1.5409

750	10.631	Palmitic acid	HMDB0000220	C16H32O2	256.2395	256.2402	3	-	-0.64896	2.342
655	6.848	3-Oxotetradecanoic acid	HMDB0010730	C14H26O3	242.1874	242.1882	3	-	-0.63426	1.6505
941	7.485	Androstenedione	HMDB0000053	C19H26O2	286.2138	286.1933	15	-	-0.60945	1.3798
1473	8.412	5alpha-Tetrahydrocortisol	HMDB0000526	C21H34O5	366.2373	366.2406	9	-	-0.60048	2.7446
411	1.282	L-Acetylcarnitine	HMDB0000201	C9H17NO4	203.1146	203.1158	6	-	0.60093	1.6219
1657	5.925	9-Hydroperoxyoctadeca-10,12-dienoic acid	HMDB0062434	C18H32O4	312.2314	312.2301	4	+	0.61029	2.0453
752	1.171	Dopa	HMDB0000181	C9H11NO4	197.0696	197.0688	4	+	0.62342	1.8085
1121	3.158	Leucylleucine	HMDB0028933	C12H24N2O3	244.1797	244.1787	4	+	0.63125	2.3275
856	3.431	Cyclo(leucylprolyl)	HMDB0034276	C11H18N2O2	210.1377	210.1368	4	+	0.72814	1.5588
636	5.454	3-Carboxy-4-methyl-5-propyl-2-furanpropionic acid	HMDB0061112	C12H16O5	240.0988	240.0998	4	-	0.91217	1.5211
2297	1.292	Estrone glucuronide	HMDB0004483	C24H30O8	446.1971	446.1941	7	+	0.91597	1.7793
2248	10.47	alpha-Tocopherol	HMDB0001893	C29H50O2	430.3782	430.3811	7	+	1.0656	1.3103
2280	9.065	3-Hydroxyoctadecenoylcarnitine	HMDB0013339	C25H47NO5	441.3468	441.3454	3	+	1.1093	1.8295
271	0.846	Iditol	HMDB0011632	C6H14O6	182.0776	182.079	8	-	1.2339	1.4268
2161	4.105	Alclometasone	HMDB0014385	C22H29ClO5	408.1736	408.1704	8	+	5.7414	1.6704
